# A nomogram for predicting three or more axillary lymph node involvement before breast cancer surgery

**DOI:** 10.1038/s41598-022-16538-z

**Published:** 2022-07-15

**Authors:** Young-Joon Kang, Jung Hyun Park, Young Wook Ju, Kyoung-Eun Kim, Yumi Kim, Eunshin Lee, Han-Byoel Lee, Dong-Young Noh, Wonshik Han

**Affiliations:** 1 Department of Surgery, College of Medicine, Incheon St. Mary’s Hospital, The Catholic University of Korea, Incheon, Republic of Korea; 2 Department of Surgery, Seoul National University College of Medicine, 101 Daehak-ro, Jongno-gu, Seoul, 03080 Republic of Korea; 3 Busan Cancer Center, Pusan National University Hospital, Busan, Republic of Korea; 4 Department of Surgery, CHA Gangnam Medical Center, CHA University School of Medicine, Seoul, Republic of Korea; 5 Division of Breast and Endocrine Surgery, Department of Surgery, Korea University College of Medicine, Seoul, Republic of Korea; 6 Laboratory of Breast Cancer Biology, Cancer Research Institute, Seoul National University College of Medicine, Seoul, Republic of Korea; 7 Seoul National University College of Medicine, Seoul, Republic of Korea

**Keywords:** Breast cancer, Cancer imaging, Cancer therapy

## Abstract

Based on the American College of Surgeons Oncology Group (ACOSOG)-Z0011, a useful nomogram has been constructed to identify patients who do not require intraoperative frozen sections to evaluate sentinel lymph nodes in the previous study. This study investigated the developed nomogram by ultrasonography (US) and positron emission tomography (PET)/computed tomography (CT) as a modality. In the training set, 89/1030 (8.6%) patients had three or more positive nodes. Larger tumor size, higher grade ultrasonographic ALN classification, and findings suspicious of positive ALN on PET/CT were associated in multivariate analysis. The areas under the receiver operating characteristic curve (AUC) of the nomogram were 0.856 [95% CI 0.815–0.897] in the training set. The AUC in the validation set was 0.866 [95% CI 0.799–0.934]. Application of the nomogram to 1067 patients who met the inclusion criteria of ACOSOG-Z0011 showed that 90 (8.4%) patients had scores above the cut-off and a false-negative result was 37 (3.8%) patients. And the specificity was 93.8%, and the negative predictive value was 96.4%. The upgraded nomogram improved the predictive accuracy, using only US and PET/CT. This nomogram is useful for identifying patients who do not require intraoperative analysis of sentinel lymph nodes and considering candidates for identifying neoadjuvant chemotherapy. The patients consisted of clinical T1-2 and node-negative invasive breast cancer. The training and validation set consisted of 1030 and 781 patients, respectively. A nomogram was constructed by analyzing factors related to three or more axillary lymph node metastases. The patients who matched the ACOSOG-Z0011 criteria were selected and applied to the new nomogram.

## Introduction

Over the past two decades, evidence from randomized clinical trials has enabled the de-escalation of axillary surgery in the management of early breast cancer^1^, with the major change has begun the replacement of axillary lymph node dissection (ALND) by sentinel lymph node biopsy (SLNB). Three randomized trials, The American College of Surgeons Oncology Group (ACOSOG)-Z0011 trial, the After Mapping of the Axilla: Radiotherapy Or Surgery (AMAROS) trial, and the International Breast Cancer Study Group (IBCSG) 23–01 trial found that complete ALND could be safely omitted^2–4^.

The ACOSOG-Z0011 trial found that complete ALND did not improve locoregional recurrence-free survival rates in clinical T1-2N0M0 breast cancer patients with 1 or 2 positive sentinel lymph nodes (SLNs) who underwent breast-conserving surgery followed by whole breast radiotherapy, thus changing the standard approach to axillary surgery^2^. Intraoperative pathology assessment of SLNs is highly effective in detecting patients who may benefit from ALND at the same surgical procedure^5^. Nevertheless, a significant proportion of these patients may not need ALND, reducing the usefulness of intraoperative pathologic examination of frozen sections. Intraoperative diagnostic techniques, including frozen sections, add to the cost and complexity of the surgery and complicate surgical scheduling. Application of the criteria derived from these studies has reduced the need to identify lymph node status intraoperatively, thus reducing the performance of ALND and pathologic assessment of frozen sections of SLNs^6^.

Less invasive techniques are needed to predict the state of SLNs before surgery. A valuable nomogram to identify patients who do not require intraoperative frozen sections to evaluate SLNs was made in the previous study^7^. That nomogram did not predict the involvement of each ALN but identified patients with ≥ 3 metastatic axillary lymph nodes (ALNs). Patients with a high probability of having ≥ 3 metastatic ALNs could be identified using preoperative imaging modalities, such as computed tomography (CT) and ultrasonography (US), as well as patient demographics and characteristics. ^18^F-fluorodeoxyglucose (FDG) positron emission tomography (PET/CT) could not be included in the previous nomogram due to the results of PET/CT having a low positive predictive value (27.2%)^7^.

Current clinical guidelines and several retrospective studies have indicated that bone scan or CT scan of chest-abdomen-pelvis (CTCAP) is not clinically useful in breast cancer staging. Despite guidelines and limited clinical evidence, these imaging modalities have been commonly used for staging work-up in patients with early breast cancer in some areas, including Asia^8–10^. As neoadjuvant systemic therapy has been widely applied, the use of these imaging modalities seems to continue in practice. PET/CT is also not recommended for asymptomatic early breast cancer patients. PET/CT scanning can identify regional nodal disease and distant metastases, suggesting that PET/CT can replace multiple imaging modalities for breast cancer staging, including axilla assessment, bone scan, and CTCAP^11^. Chest CT can evaluate limited areas for metastases, whereas PET/CT can evaluate the entire body. Moreover, PET/CT is highly specific regarding regional lymph node involvement^12^ and has been used more than chest CT for evaluating ALN metastases, despite lacking sensitivity^13^. Recently, Hyland represented that PET/CT reduced the risk of false-positive in half and reduced work-up for incidental findings, enabling early treatment initiation^14^.

The previous study enrolled patients who underwent surgery between 2006 and 2011, a period during which only 19.9% of these patients underwent ^18^F-FDG PET/CT. These provided another reason for excluding ^18^F-FDG PET/CT results from the previous nomogram. This study enrolled patients who had sufficient PET/CT images from a more recent period. And using this, it aimed to create a more improved nomogram that predicts the probability of ≥ 3 ALNs.

## Results

Patient and tumor characteristics, as well as treatment and preoperative imaging findings, are shown in Table 1. The mean age at diagnosis was 51.4 years (range, 24–82 years). Of the 1030 patients in the training set, 89 (8.6%) showed involvement of ≥ 3 ALNs, and 295 (28.6%) underwent ALND. ^18^F-FDG PET/CT findings were available for 853 patients (82.8%), with 188 (22.0%) of these patients having findings suspicious of ALN metastasis.

**Table 1:** Patient characteristics of the training set (N = 1030).

Characteristics	No	%
**Age (years)**		
Mean	51.4 ± 10.4	
Range	24–82	
**Tumor size by pre-op US (cm)**		
Mean	2.1 ± 1.0	
Range	0.3–5.0	
**Axillary LN involvement**		
≤ 2	941	91.4
≥ 3	89	8.6
**Axillary LN classification**		
1	343	33.3
2	378	36.7
3	168	16.3
4	63	6.1
5	59	5.7
Unknown	19	1.8
**PET/CT-ALN (SUV)**		
Positive (≥ 1.4)	188	18.3
Negative (< 1.4)	665	64.6
Unknown	177	17.2
**Surgery-breast**		
Conservation	768	74.6
Mastectomy	262	25.4
**Surgery-axilla**		
Sentinel LN biopsy	735	71.4
ALND	295	28.6
**Pathology**		
Ductal	964	93.6
Lobular	66	6.4
Other		
**Pathologic tumor size**		
≤ 2 cm	572	55.5
> 2 cm	458	44.5
**Estrogen receptor**		
Positive	791	76.8
Negative	224	21.7
Unknown	15	1.5
**Progesterone receptor**		
Positive	661	64.2
Negative	358	34.8
Unknown	11	1.1
**HER2 receptor**		
Positive	264	19.6
Negative	563	54.7
Unknown	202	19.6

*Pre-op*, Preoperative; *US*, Ultrasonography; *LN*, Lymph node; *ALN*, Axillary lymph node; *PET/CT*, Positron emission tomography/computed tomography; *ALND*, Axillary lymph node dissection; *HER2*, Human epidermal growth factor receptor 2.

Univariate logistic regression analysis (Table 2) showed that having ≥ 3 ALNs was significantly associated with patient age (*p* = 0.048); mean tumor size on US (*p* < 0.001) and magnetic resonance imaging (MRI) (*p* < 0.001); tumor stage on US (*p* < 0.001); axillary ultrasonographic ALN classification (*p* < 0.001); chest CT findings suspicious of positive ALN (*p* < 0.001); and PET/CT findings suspicious of positive ALN (*p* < 0.001).

**Table 2:** Univariate logistic regression analysis for factors associated with involvement of three or more axillary lymph nodes.

Characteristics	3 or more LN( +) (%)	2 or less LN( +) (%)	*p*-value
Age (years), mean	51.5	50.6	0.048
Mean tumor size by US (cm)	2.83 ± 1.07	2.05 ± 1.01	< 0.001
Mean tumor size by MRI (cm)	3.25 ± 1.47	2.38 ± 1.41	< 0.001
Tumor stage by US (cm)			< 0.001
T1 (≤ 2)	541 (57.5)	25 (28.1)	
T2 (> 2–5)	400 (42.5)	64 (71.9)	
Axillary US classification			< 0.001
Gr 1	5 (5.7)	338 (36.6)	
Gr 2	16 (18.4)	362 (39.2)	
Gr 3	17 (19.5)	151 (16.3)	
Gr 4	24 (27.6)	39 (4.2)	
Gr 5	25 (28.7)	34 (3.7)	
PET/CT-ALN (SUV)			< 0.001
Positive (≥ 1.4)	52 (61.9)	136 (17.7)	
Negative (< 1.4)	32 (38.1)	633 (82.3)	
Estrogen receptor			0.716
Positive	68 (76.4)	723 (78.1)	
Negative	21 (23.6)	203 (21.9)	
Progesterone receptor		0.853	
Positive	55 (64.0)	606 (65.0)	
Negative	31 (36.0)	327 (35.0)	
HER2 receptor		0.659	
Positive	23 (34.3)	241 (31.7)	
Negative	44 (65.7)	519 (68.3)	

Values are presented as mean ± standard deviation or number (%). Abbreviations: *ALN*, Axillary lymph node; *LN*, Lymph node; *US*, Ultrasonography; *Gr*, Grade; *MRI*, Magnetic resonance imaging; *PET/CT*, Positron emission tomography/computed tomography; *HER2*, Human epidermal growth factor receptor 2.

Multivariate logistic regression analyses (Table 3) were performed using as factors patient age, tumor size on preoperative US (cm), ultrasonographic ALN classification, and PET/CT findings suspicious of positive ALN. Chest CT and MRI were significant factors in the univariate analysis but were excluded from multivariate analysis. Tumor size on MRI could be controversial to evaluate uniformly. These analyses showed that larger tumor size (odds ratio [OR], 1.58; 95% confidence interval [CI], 1.23–2.01; *p* < 0.001), higher grade ultrasonographic ALN classification (OR 2.03; 95% CI 1.61–2.56; *p* < 0.001) and PET/CT findings suspicious of positive ALN (OR 2.64; 95% CI 1.47–4.75; *p* = 0.001) were significant and independent predictors of having ≥ 3 involved ALNs.

**Table 3:** Multivariate logistic regression analysis for factors associated with involvement of three or more axillary lymph nodes.

Variable	Odds ratio	95% CI	*p*-value
Age	0.99	0.97–1.02	0.636
Tumor size by pre-op US (cm)	1.58	1.23–2.01	< 0.001
Axillary US grade	2.03	1.61–2.56	< 0.001
PET/CT-ALN positive (SUV)	2.64	1.47–4.75	0.001

*ALN*, Axillary lymph node; *CI*, Confidence interval; *US*, Ultrasonography; *PET/CT*, Positron emission tomography/computed tomography.

**Figure 1:**
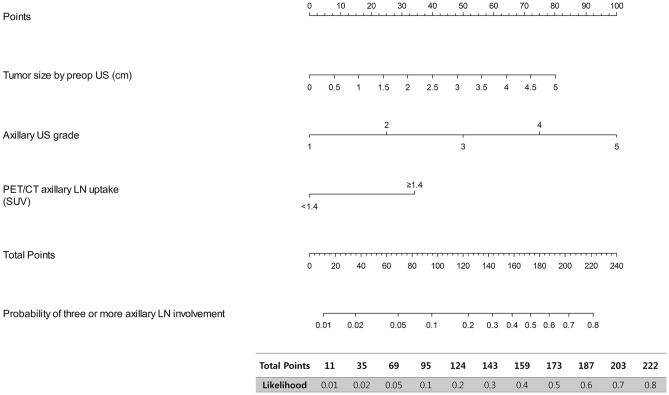
Nomogram for predicting the probability of having three or more involved axillary lymph nodes. Abbreviations: US, ultrasonography; PET/CT, Positron emission tomography/computed tomography.

**Figure 2:**
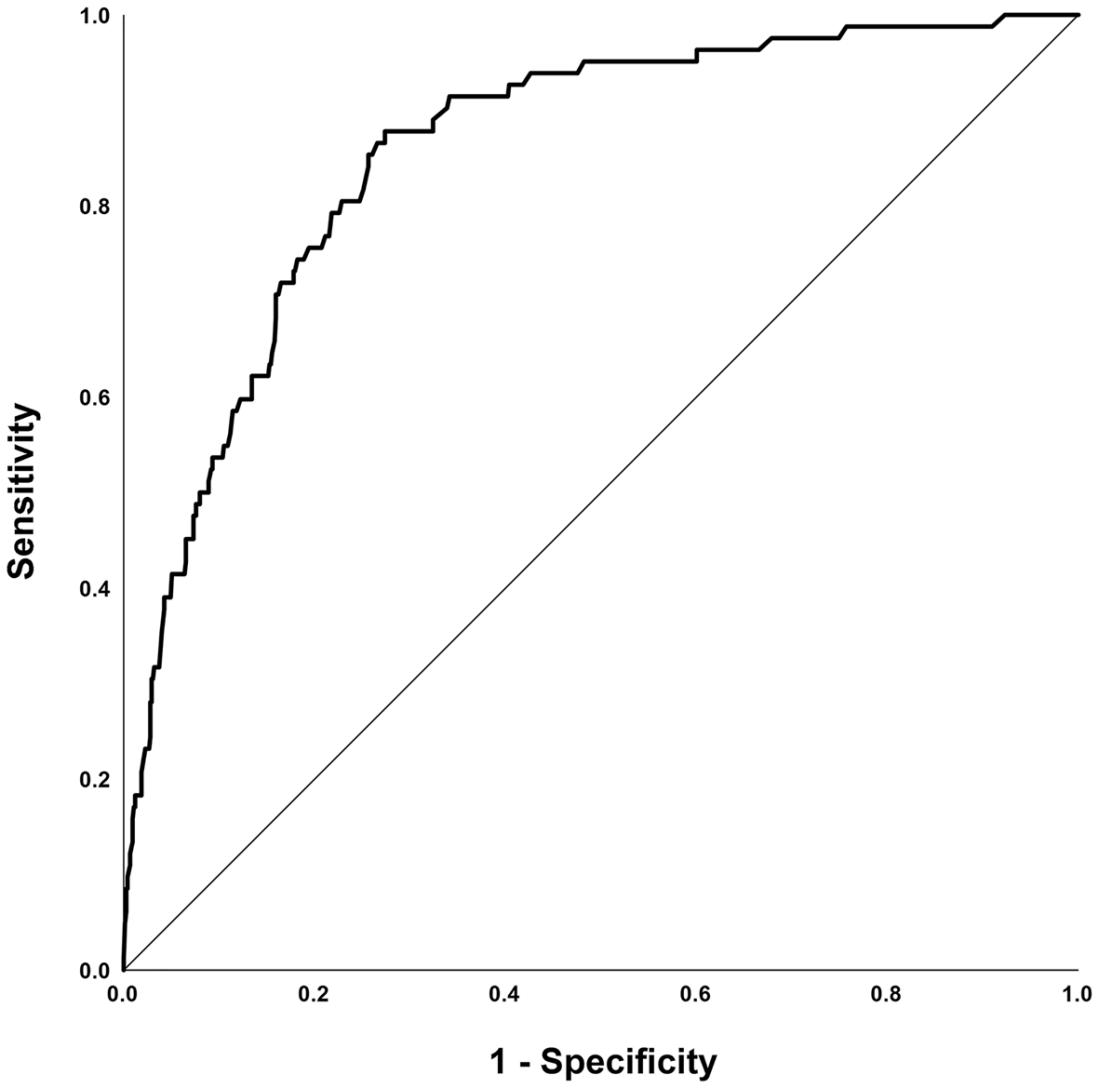
The performance of the nomogram in the training set was measured using the area under the receiver operating characteristic curve (AUC). AUC of the training set: 0.856 (95% confidence interval, 0.815 to 0.897).

**Figure 3:**
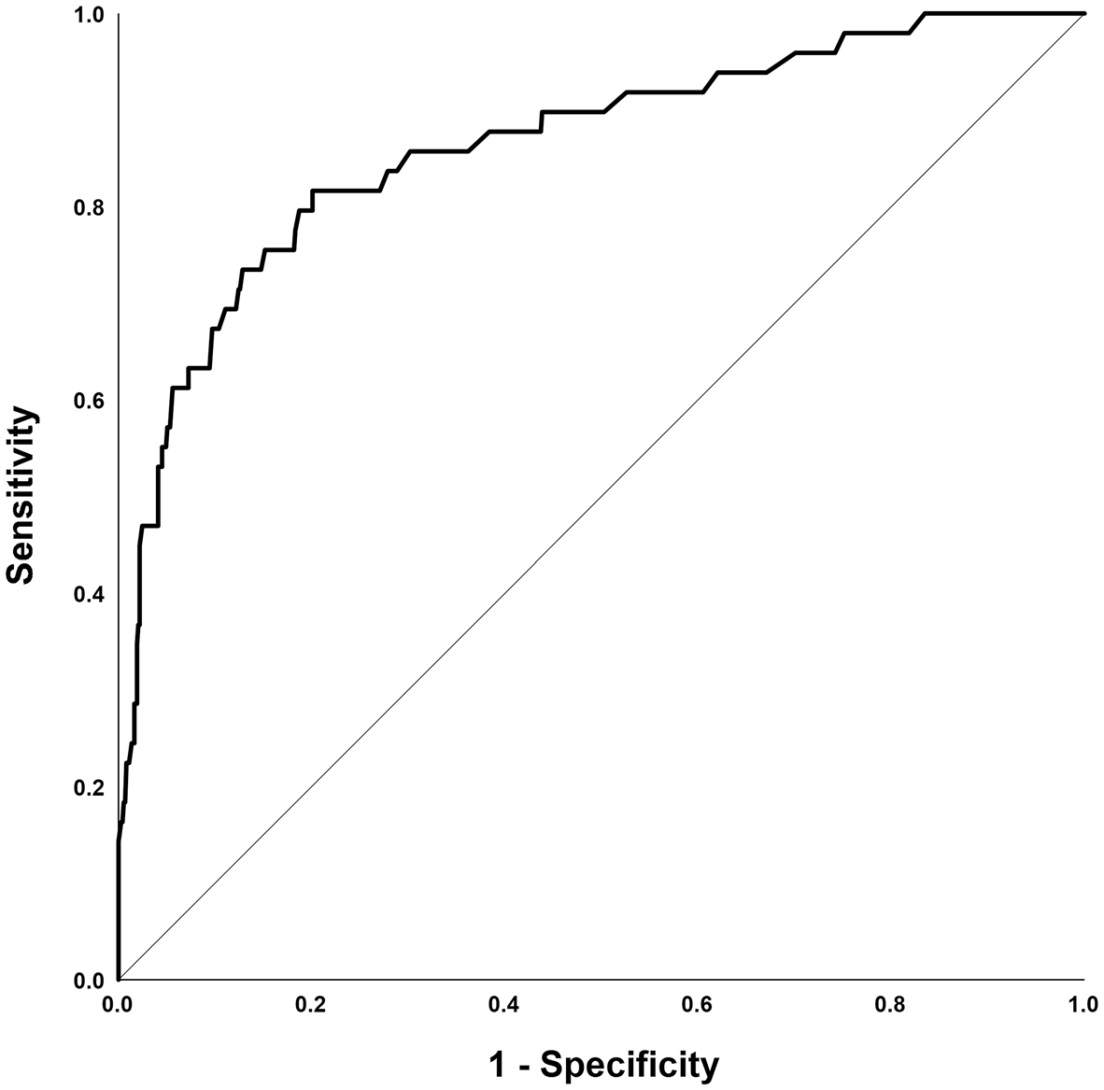
The performance of the nomogram in the validation set was measured using the area under the receiver operating characteristic curve (AUC). AUC of the validation set: 0.866 (95% confidence interval, 0.799 to 0.934).

**Figure 4:**
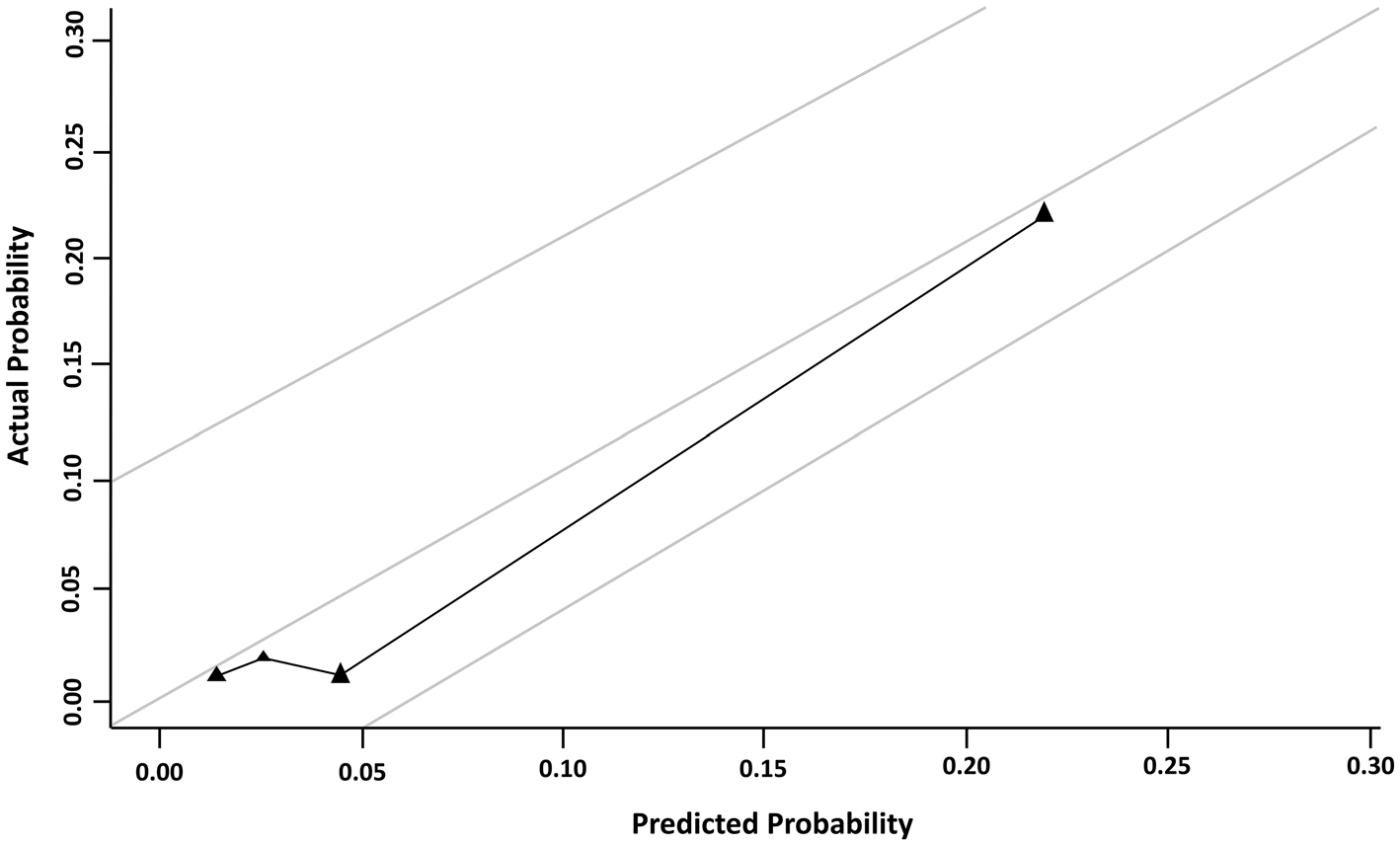
Calibration plot of the nomogram using validation cohort.

The results of this multivariate analysis were used to construct a nomogram predicting the probability of involvement of ≥ 3 positive ALNs. To account for the method of constructing the developed nomogram by giving points to each variable, tumor size by preoperative US, axillary US grade, and PET/CT axillary LN uptake. Then, they were summed to give the total number of points. The factors were used to assign a probability of ≥ 3 positive ALNs for each patient using the scale at the bottom of Fig. 1. The ROC curve of the nomogram was plotted, and the cut-off number of points was used in R statistics. The optimum cut-off was 120 points, which yielded an AUC of 0.856 (95% CI, 0.815–0.897) for the training set (Fig. 2). Table 4 shows the characteristics of the 781 patients in the validation set. Their mean age at diagnosis was 51.9 years, 49 (6.3%) patients had ≥ 3 ALNs, and 137 (17.5%) underwent ALND. The AUC of the validation set was 0.866 (95% CI, 0.799–0.934) (Fig. 3). The actual probability of involvement of ≥ 3 positive ALN for each patient in the validation set was plotted against the calculated predicted probability of ≥ 3 positive ALN to evaluate the accuracy of the nomogram (Fig. 4). The overall predictive accuracy of the model was within an error range of 10%.

**Table 4:** Patient characteristics of the validation set (N = 781).

Characteristics	No	%
**Age (years)**		
Mean	51.9 ± 10.4	
Range	25–85	
**Tumor size by pre-op US (cm)**		
Mean	2.0 ± 1.0	
Range	0.5–5.0	
Axillary LN involvement		
≤ 2	732	93.7
≥ 3	49	6.3
**Axillary LN classification**		
1	258	33.0
2	328	42.0
3	106	13.6
4	46	5.9
5	33	4.2
Unknown	10	1.3
**PET/CT-ALN (SUV)**		
Positive (≥ 1.4)	94	12.0
Negative (< 1.4)	586	75.0
Unknown	101	12.9
Surgery-breast		
Conservation	516	66.1
Mastectomy	265	33.9
Surgery-axilla		
Sentinel LN biopsy	644	82.5
ALND	137	17.5
**Pathology**		
Ductal	740	94.8
Lobular	41	5.2
**Pathologic tumor size**		
≤ 2 cm	509	65.2
> 2 cm	272	34.8
**Estrogen receptor**		
Positive	609	78.0
Negative	164	21.0
Unknown	8	1.0
**Progesterone receptor**		
Positive	513	65.7
Negative	259	33.2
Unknown	9	1.1
**HER2 receptor**		
Positive	102	13.1
Negative	620	79.4
Unknown	59	7.5

*Pre-op*, Preoperative; *US*, Ultrasonography; *LN*, Lymph node; *ALN*, Axillary lymph node; *PET/CT*, Positron emission tomography/computed tomography; *ALND*, Axillary lymph node dissection; *HER2*, Human epidermal growth factor receptor 2.

Table 5 shows the accuracy of the nomogram in predicting the involvement of ≥ 3 ALNs by the calculated specificity and negative predictive value (NPV). This analysis excluded patients with predictive factors that could not be measured, including 195 patients in the training set and 109 in the validation set. The nomogram had a specificity of 89.2% and an NPV of 94.7% using a cut-off of 120 points in the training set. This nomogram yielded false-negative results in 37 (5.2%) patients, who were predicted by the nomogram to have ≤ 2 rather than ≥ 3 LNs. In addition, the nomogram indicated that 126 (15.1%) patients in the training set required intraoperative assessment of frozen sections of SLNs. When applied to the validation set, the nomogram had a specificity of 93.0% and an NPV of 97.2%, and false-negative results were obtained in 16 (2.6%) patients, who were predicted by the nomogram to have ≤ 2 rather than ≥ 3 LNs. The nomogram indicated that 65 (9.7%) patients in the validation set required intraoperative assessment of frozen sections of SLNs.

**Table 5:** Two-way contingency table analysis showing predictive accuracy of the nomogram.

		Training set			Validation set		
		Observed (N)			Observed (N)		
		LN ≥ 3	LN ≤ 2	Total	LN ≥ 3	LN ≤ 2	Total
Expected (N)	LN ≥ 3	45	81	126	27	38	65
	LN ≤ 2	37	672	709	16	591	607
	Total	82	753	835	43	629	672
Accuracy % (95% CI)		85.8 (0.83–0.88)			90.9 (0.89–0.93)		
Sensitivity % (95% CI)		54.9 (0.44–0.65)			59.5 (0.45–0.74)		
Specificity % (95% CI)		89.2 (0.87–0.91)			93.0 (0.91–0.95)		
PPV % (95% CI)		35.7 (0.29–0.42)			36.2 (0.28–0.45)		
NPV % (95% CI)		94.7 (0.94–0.96)			97.2 (0.96–0.98)		

*LN*, Lymph node; *PPV*, Positive predictive value; *NPV*, Negative predictive value; *CI*, Confidence interval.

The nomogram was also applied to 1067 patients who met the selection criteria of the ACOSOG-Z0011 trial (Table 6). The nomogram had a specificity of 93.8%, and an NPV was 96.4% using a cut-off of 120 points. Of the 1067 patients predicted to have ≤ 2 metastatic ALNs, only 37 (3.8%) showed false-negative results. Ninety (8.4%) patients had nomogram scores above the cut-off and required intraoperative assessment of frozen sections of SLNs.

**Table 6:** Predictive accuracy of the nomogram in patients who meet the ACOSOG-Z0011 criteria.

		Observed (N)		
		LN ≥ 3	LN ≤ 2	Total
Expected (N)	LN ≥ 3	27	63	90
	LN ≤ 2	37	940	977
	Total	64	1003	1067
Accuracy % (95% CI)		90.8 (0.89–0.92)		
Sensitivity % (95% CI)		45.3 (0.33–0.58)		
Specificity % (95% CI)		93.8 (0.92–0.95)		
PPV % (95% CI)		31.9 (0.25–0.40)		
NPV % (95% CI)		96.4 (0.96–0.97)		

*LN*, Lymph node; *PPV*, Positive predictive value; *NPV*, Negative predictive value; *CI*, Confidence interval.

## Discussion

This upgraded nomogram is useful for identifying patients who do not require intraoperative analysis of sentinel lymph nodes. And it improved the predictive accuracy. In contrast to the previous nomogram based on chest CT results, the current nomogram included ^18^F-FDG PET/CT results. In practice, this nomogram improved the predictive accuracy, with a specificity of 93.8% and an NPV of 96.4%.

Omission of ALND in women who satisfy the eligibility of the ACOSOG-Z0011 trial is a major step in reducing the intensity of surgery and the burden of treatment. The percentage of patients undergoing an intraoperative examination of frozen sections of SLNs also has been declining for over a decade^15^. Intraoperative evaluation of SLN decreased from 69 (230/335) to 26% (84/323) and from 92 (54/59) to 45% (14/31), according to observational studies which investigated the changes in medical practice after publishing the ACOSOG-Z0011 trial^16^. The role of frozen sections remains unclear^17^. Frozen sections have several drawbacks, including increased operation time. Although the SLNB procedure is not time-consuming, the turnaround time for evaluating intraoperative frozen sections is considerable compared to the total operation time. Breast cancer surgery often requires analyzing many tissues with the patient remaining anesthetized until the pathologic outcome is determined. Because the benefit of avoiding reoperative ALND is only 12.4% of patients, routine frozen sections may be indicated for only a selected group of patients, such as those with larger tumors^18^. Additionally, the process of obtaining intraoperative frozen sections can risk the destruction of potentially diagnostic tissue. The quality of frozen tissues may not be as high as well-fixed tissue preparations, and incomplete sections may exclude the critical subcapsular sinus^6^. Prior freezing may compromise the quality of paraffin section histology^19^.

Although noninvasive methods and preoperative imaging modalities have been evaluated^20–22^, their feasibility was insufficient to replace intraoperative frozen sections in the evaluation of SLNs. We previously reported that US classification had a sensitivity of 85% and a specificity of 78% in predicting ALN metastasis with an AUC of 0.861 (95% CI, 0.796–0.926)^23^. A review of 21 studies evaluating preoperative US-guided needle biopsy reported that the median sensitivity was 64% and the median specificity was 82%^24^. In a previous study, a prospective evaluation of 512 patients who met the criteria of ACOSOG-Z0011 trial and underwent surgery at our institution from January 2012 to June 2014 found that the intraoperative frozen section for evaluation of SLNs could be omitted in 452 (88.3%) cases. The reoperation rate (final pathology ≥ 3 LNs) was 1.6% (8/512) when the score was low and intraoperative frozen sectioning was not performed^7^. Recently, neoadjuvant chemotherapy has been increasingly used for early breast cancer. It may be possible to consider applying neoadjuvant chemotherapy in patients who would be predicted over three positive LNs by this nomogram depending on the subtype.

This method also has several disadvantages. First, ^18^F-FDG PET/CT results are required to apply the nomogram. Although several benefits have been mentioned for the use of PET/CT in early-stage breast cancer, in clinical practice, many clinicians do not schedule preoperative PET/CT in patients with early breast cancer. Recently, a study has revealed that preoperative PET/CT could predict high nodal burden with high accuracy. It appears that preoperative PET/CT is useful to perform before developing a treatment plan for patients with clinical T1-2N0 invasive breast cancer^25^. Additionally, the NCCN guidelines in 2021 recommend that FDG PET/CT can be performed concurrently with diagnostic CT as a work-up prior to preoperative systemic therapy if clinical T2 or clinical node-positive. In these situations, it will be possible that this nomogram can be used as one of the clinical bases for using PET/CT in early-stage breast cancer. When the patients enrolled in this study were diagnosed, they had little financial burden (5% deductible) because national insurance was applied, so most of them were taken the PET/CT. Although it is not a guideline at present, it might be one of the data for replacing the multiple CT scan of chest-pelvis-abdomen with PET/CT in regions frequently used in early breast cancer. Second, the application of this nomogram requires an experienced US operator^23^. The US operator measures tumor size and classifies the probability of lymph node metastasis based on the thickness of the cortex and the appearance of the fatty hilum. Contrary to MRI, the US depends on the patient body habitus and the operator's experience^26^. The US finding for nodal status in this study was applied to grade according to the maximum thickness of the cortex and the appearance of the fatty hilum. Therefore, it depended on the US operator and required an experienced US operator^23^. Third, the results of MRI were not included in the nomogram. MRI features of breast cancer can help in its diagnosis, making it a frequently used imaging modality in these patients. Non-mass enhancement on MRI may increase suspicion of an invasive lesion, particularly if the enhancement is associated with a focal lesion or exhibits a segmental distribution^27^. Although univariate analysis showed that larger tumor size on MRI was significantly associated with the involvement of ≥ 3 ALNs, this parameter could be replaced by tumor size in US.

According to the previous and this study, when PET/CT is taken, this nomogram applies to the patients, and for patients without PET/CT, chest CT is used to calculate the previous nomogram. If further research related to this is conducted, it is expected that taking PET/CT will be more advantageous.

In conclusion, we made a nomogram based on preoperative imaging modalities that can predict the involvement of ≥ 3 ALNs in women with early-stage breast cancer. This nomogram had excellent predictive power and was clinically useful. Intraoperative frozen sections for the detection of SLNs could be omitted in a significant number of patients who met the criteria of the ACOSOG-Z0011 trial, with a low rate of reoperation. This method could evaluate the pathology of SLNs in permanent sections, providing more accurate pathologic results, simplified surgical scheduling, saving time and costs. This is useful for identifying patients who do not require intraoperative analysis of SLNs. And, it would be considered that indications for applying neoadjuvant chemotherapy can be made through further studies based on this nomogram. This nomogram can be applied with the other purpose for luminal A and postmenopausal patients. It can select those who will proceed to upfront surgery rather than neoadjuvant chemotherapy if the patient has genomic low risk with multigene assay and limited number of involved LNs is predicted by our nomogram. Moreover, this study could be regarded as one of the backgrounds that PET/CT can replace multiple imaging modalities for preoperative work-up. This processing allows omitting unnecessary ALN assessment during operation.

## Methods

### Patients

The Seoul National University Hospital Breast Cancer Center (SNUHBCC) database, a relatively large, prospectively maintained web-based database that includes information on all patients who have undergone surgery for breast disease at Seoul National University Hospital since 1982 was reviewed. Detailed information regarding the SNUHBCC database that was prospectively collected after obtaining institutional review board approval has been reported^28^.

Patients who had clinical T1-2 and N0 invasive breast cancer and underwent preoperative ^18^F-FDG PET/CT were included. The training set included 1030 consecutive patients who underwent surgery between 2010 and 2013, whereas the validation set included 781 patients who underwent surgery from 2014 to 2015. Patients with a history of breast cancer, palpable ALNs, or carcinoma in situ on preoperative core needle biopsy were excluded, as were patients who received neoadjuvant chemotherapy, those with tumors over 5 cm on the preoperative US, and patients with stage IV breast cancer. Because the ACOSOG-Z0011 trial set the patients who underwent breast-conserving surgery as inclusion criteria, it was necessary to analyze patients who met the criteria to predict how clinically accurate the nomogram would be. After validating the nomogram, 1067 patients who met the inclusion criteria of the ACOSOG-Z0011 trial were selected and analyzed from the training and validation set.

### Preoperative imaging

All patients underwent US and ^18^F-FDG PET/CT for preoperative work-up of the axilla and distant organs. All images were reported by experienced radiologists who had received information that the patients were diagnosed with invasive breast cancer. ALNs were evaluated one day before surgery by axillary US examination. Lymph nodes were prospectively classified by a radiologist. The probability of lymph node metastasis on the US was classified according to the maximum thickness of the cortex and the appearance of fatty hilum, with grade 1 indicating cortical thickness of ≤ 1.5 mm; grade 2 indicating 1.5 mm < cortical thickness ≤ 2.5 mm; grade 3 indicating 2.5 mm < cortical thickness ≤ 3.5 mm; grade 4 indicating cortical thickness > 3.5 mm with an intact fatty hilum; and grade 5 indicating cortical thickness > 3.5 mm with a loss of fatty hilum. The maximum cortical thickness was measured perpendicular to the long axis of the lymph node on a cross-sectional plane^23^.

^18^F-FDG PET/CT imaging was taken using a hybrid PET/CT scanner (Biograph 40 TruePoint; Siemens Healthcare, Knoxville, TN, USA). The patients were fasted for at least 6 h prior to being administered ^18^F-FDG (5.18 MBq/kg of body weight, intravenously), and imaging was performed 1 h later. CT images were acquired from the skull base to the upper thigh area for the attenuation map and lesion localization (50 mA, 120kVp, 5-mm section width, 4 mm collimation). After CT scanning, PET images of the same area were acquired in three-dimensional mode at six or seven-bed positions (1 min per bed position, 21.6 cm increments). Images were reconstructed on 128 × 128 matrices using an iterative algorithm. The PET/CT images were analyzed using a dedicated workstation and analysis software (Syngo.via, Siemens Healthcare) and interpreted by institutional nuclear medicine physicians individually as a standard-of-care examination. Positivity was defined as the PET/CT ALN uptake SUV set to 1.4 or over.

### Management of ALNs

SLNs were detected intraoperatively using the SLNB technique, a radioisotope, and/or a blue dye. Alternatively, Tc-99 m antimony sulfur colloid (0.4 mCi) was intradermally injected 1 to 6 h before surgery into the quadrant in which the tumor was located. Lymphoscintigraphic images were attained about 40 min after injection, and SLNs were detected during operation by a gamma probe (NEO2000; Neoprobe Co., Dublin, USA). Immediately before surgery, 1 cc aliquots of 0.8% indigo carmine dye were injected intradermally into four subareolar areas around each areola. SLNs were defined as nodes with the hottest node, and any other nodes increased radioactivity at least 10% of the hottest node by the gamma probe and/or stained with blue dye. SLNs and suspicious metastatic nodes in the surgical field were removed; most were bisected and examined by hematoxylin and eosin staining of frozen sections during the surgery. ALND was performed when malignant cells were found in one or more sentinel lymph nodes. Postoperatively, SLNs were fixed in formalin, embedded in paraffin, and sectioned at 4 µm thickness. Patients with positive SLNBs in the training and validation underwent complete level I and II ALND.

### Statistical analysis

The associations of ≥ 3 involved SLNs with patient demographic characteristics, tumor characteristics on biopsy, and preoperative work-up imaging results were evaluated by Fisher’s exact test. Multivariate logistic regression analyses were performed using combinations of continuous variables (age, tumor size on US, and ultrasonographic ALN classification) and dichotomized variables (positivity of ALN on PET/CT). A nomogram was generated based on a multivariate logistic regression model that predicted the probability of involvement of ≥ 3 ALNs. A forward stepwise selection method and likelihood ratio test were used to select a subgroup of all analyzed factors.

The performances of the nomogram were assessed using receiver operating characteristic (ROC) curve analysis and calculation of the areas under the ROC curves (AUCs). Calibration of the nomogram was evaluated by plotting the observed probabilities against the predicted probabilities calculated with the nomogram. A perfectly accurate nomogram prediction model would produce a plot where the observed and predicted probabilities for given groups fall along the 45-degree line. The distance between the pairs and the 45-degree line is a measure of the absolute error of prediction of the nomogram^29^. All statistical analyses were performed using SPSS ver. 21.0 (SPSS Inc., Chicago, IL) and R Software ver. 3.6.3 (http://www.r-project.org/), with *p* < 0.05 considered statistically significant.

### Ethics approval and consent to participate

This study was approved by the Institutional Review Board of the Catholic Medical Center (IRB no. OC21EISI0076) and was conducted in accordance with the tenets of the Declaration of Helsinki. The requirement for informed consent was waived.

## Data Availability

The demographic and clinical data collected for the purpose of the statistical analysis to support the findings of this study are available from the corresponding author upon request.

## Abbreviations

ACOSOG: American College of Surgeons Oncology Group
ALN: Axillary lymph node
ALND: Axillary lymph node dissection
AUC: Areas under the receiver operating characteristic curve
CT: Computed tomography
CTCAP: CT scan of chest-abdomen-pelvis
FDG: ^18^F-fluorodeoxyglucose
MRI: Magnetic resonance imaging
NPV: Negative predictive value
PET: Positron emission tomography
ROC: Receiver operating characteristic
SLN: Sentinel lymph node
SLNB: Sentinel lymph node biopsy
SLNUHBCC: Seoul National University Hospital Breast Cancer Center
US: Ultrasonography

## References

[1] Valero MG, Golshan M (2018). Management of the axilla in early breast cancer. Cancer Treat. Res..

[2] Giuliano AE, Hunt KK, Ballman KV, Beitsch PD, Whitworth PW, Blumencranz PW, Leitch AM, Saha S, McCall LM, Morrow M (2011). Axillary dissection vs no axillary dissection in women with invasive breast cancer and sentinel node metastasis: A randomized clinical trial. JAMA.

[3] Donker M, van Tienhoven G, Straver ME, Meijnen P, van de Velde CJ, Mansel RE, Cataliotti L, Westenberg AH, Klinkenbijl JH, Orzalesi L (2014). Radiotherapy or surgery of the axilla after a positive sentinel node in breast cancer (EORTC 10981–22023 AMAROS): A randomised, multicentre, open-label, phase 3 non-inferiority trial. Lancet Oncol..

[4] Galimberti V, Cole BF, Zurrida S, Viale G, Luini A, Veronesi P, Baratella P, Chifu C, Sargenti M, Intra M (2013). Axillary dissection versus no axillary dissection in patients with sentinel-node micrometastases (IBCSG 23–01): A phase 3 randomised controlled trial. Lancet. Oncol..

[5] Taffurelli M, Montroni I, Santini D, Fiacchi M, Zanotti S, Ugolini G, Serra M, Rosati G (2012). Effectiveness of sentinel lymph node intraoperative examination in 753 women with breast cancer: Are we overtreating patients?. Ann. Surg..

[6] Bishop JA, Sun J, Ajkay N, Sanders MA (2016). Decline in frozen section diagnosis for axillary sentinel lymph nodes as a result of the American college of surgeons oncology group Z0011 trial. Arch. Pathol. Lab. Med..

[7] Ahn SK, Kim MK, Kim J, Lee E, Yoo TK, Lee HB, Kang YJ, Kim J, Moon HG, Chang JM (2017). Can we skip intraoperative evaluation of sentinel lymph nodes? Nomogram predicting involvement of three or more axillary lymph nodes before breast cancer surgery. Cancer Res. Treat..

[8] National Health Commission Of The People's Republic Of C Chinese guidelines for diagnosis and treatment of breast cancer 2018 (English version). *Chin. J. Cancer Res.* **31**(2), 259–277 (2019). 10.21147/j.issn.1000-9604.2019.02.02 PMC6513740 31156298

[9] Rajasooriyar C, Sritharan T, Chenthuran S, Indranath K, Surenthirakumaran R (2020). The role of staging computed tomography on detection of occult metastasis in asymptomatic breast cancer patients. Cancer Rep. (Hoboken).

[10] Barcenas, C. H. The use of imaging and tumor markers in the staging of patients age <65 years with early-stage breast cancer. *Cancer Res.* 2013, SABCS13-P3-06-02.

[11] Ming Y, Wu N, Qian T, Li X, Wan DQ, Li C, Li Y, Wu Z, Wang X, Liu J (2020). Progress and future trends in PET/CT and PET/MRI molecular imaging approaches for breast cancer. Front. Oncol..

[12] Ueda S, Tsuda H, Asakawa H, Shigekawa T, Fukatsu K, Kondo N, Yamamoto M, Hama Y, Tamura K, Ishida J (2008). Clinicopathological and prognostic relevance of uptake level using 18F-fluorodeoxyglucose positron emission tomography/computed tomography fusion imaging (18F-FDG PET/CT) in primary breast cancer. Jpn. J. Clin. Oncol..

[13] Bellevre D, Blanc Fournier C, Switsers O, Dugue AE, Levy C, Allouache D, Desmonts C, Crouet H, Guilloit JM, Grellard JM (2014). Staging the axilla in breast cancer patients with (1)(8)F-FDG PET: How small are the metastases that we can detect with new generation clinical PET systems?. Eur. J. Nucl. Med. Mol. Imaging.

[14] Hyland CJ, Varghese F, Yau C, Beckwith H, Khoury K, Varnado W, Hirst GL, Flavell RR, Chien AJ, Yee D (2020). Use of 18F-FDG PET/CT as an initial staging procedure for stage II-III breast cancer: A multicenter value analysis. J. Natl. Compr. Canc. Netw..

[15] Weber WP, Barry M, Stempel MM, Junqueira MJ, Eaton AA, Patil SM, Morrow M, Cody HS (2012). A 10-year trend analysis of sentinel lymph node frozen section and completion axillary dissection for breast cancer: Are these procedures becoming obsolete?. Ann. Surg. Oncol..

[16] Caudle AS, Hunt KK, Tucker SL, Hoffman K, Gainer SM, Lucci A, Kuerer HM, Meric-Bernstam F, Shah R, Babiera GV (2012). American college of surgeons oncology group (ACOSOG) Z0011: Impact on surgeon practice patterns. Ann. Surg. Oncol..

[17] Cipolla C, Cabibi D, Fricano S, Vieni S, Gentile I, Latteri MA (2010). The value of intraoperative frozen section examination of sentinel lymph nodes in surgical management of breast carcinoma. Langenbecks Arch. Surg..

[18] Francissen, C. M., van la Parra, R. F., Mulder, A. H., Bosch, A. M., de Roos, W. K. Evaluation of the benefit of routine intraoperative frozen section analysis of sentinel lymph nodes in breast cancer. *ISRN Oncol.* **2013**, 843793 (2013). 10.1155/2013/843793 PMC3791598 24167745

[19] Lyman GH, Temin S, Edge SB, Newman LA, Turner RR, Weaver DL, BensonBosserman ABLD, Burstein HJ, Cody H (2014). Sentinel lymph node biopsy for patients with early-stage breast cancer: American society of clinical oncology clinical practice guideline update. J. Clin. Oncol..

[20] Nori J, Vanzi E, Bazzocchi M, Bufalini FN, Distante V, Branconi F, Susini T (2007). Role of axillary ultrasound examination in the selection of breast cancer patients for sentinel node biopsy. Am. J. Surg..

[21] Lee MC, Eatrides J, Chau A, Han G, Kiluk JV, Khakpour N, Cox CE, Carter WB, Laronga C (2011). Consequences of axillary ultrasound in patients with T2 or greater invasive breast cancers. Ann. Surg. Oncol..

[22] Peare R, Staff RT, Heys SD (2010). The use of FDG-PET in assessing axillary lymph node status in breast cancer: A systematic review and meta-analysis of the literature. Breast Cancer Res. Treat.

[23] Cho N, Moon WK, Han W, Park IA, Cho J, Noh DY (2009). Preoperative sonographic classification of axillary lymph nodes in patients with breast cancer: Node-to-node correlation with surgical histology and sentinel node biopsy results. AJR Am. J. Roentgenol..

[24] Houssami N, Ciatto S, Turner RM, Cody HS, Macaskill P (2011). Preoperative ultrasound-guided needle biopsy of axillary nodes in invasive breast cancer: Meta-analysis of its accuracy and utility in staging the axilla. Ann. Surg..

[25] Kong E, Choi J (2021). The new perspective of PET/CT for axillary nodal staging in early breast cancer patients according to ACOSOG Z0011 trial PET/CT axillary staging according to Z0011. Nucl. Med. Commun..

[26] Chang JM, Leung JWT, Moy L, Ha SM, Moon WK (2020). Axillary nodal evaluation in breast cancer: State of the art. Radiology.

[27] Macura, K. J., Ouwerkerk, R., Jacobs, M. A., Bluemke, D. A. Patterns of enhancement on breast MR images: Interpretation and imaging pitfalls. *Radiographics*, **26**(6):1719–1734 (2006); quiz 1719. 10.1148/rg.266065025 PMC5952612 17102046

[28] Moon HG, Han W, Noh DY (2009). Underweight and breast cancer recurrence and death: A report from the Korean breast cancer society. J. Clin. Oncol..

[29] Iasonos A, Schrag D, Raj GV, Panageas KS (2008). How to build and interpret a nomogram for cancer prognosis. J. Clin. Oncol..

